# Intentional and unintentional poisoning in Pakistan: a pilot study using the Emergency Departments surveillance project

**DOI:** 10.1186/1471-227X-15-S2-S2

**Published:** 2015-12-11

**Authors:** Nadeem Ullah Khan, Ricardo Pérez-Núñez, Nudrat Shamim, Uzma Rahim Khan, Naureen Naseer, Asher Feroze, Junaid Abdul Razzak, Adnan A Hyder

**Affiliations:** 1 Department of Emergency Medicine, Aga Khan University, Karachi, Pakistan; 2 International Injury Research Unit, Department of International Health, Johns Hopkins Bloomberg School of Public Health, Baltimore, MD, USA; 3 Health Systems Research Centre of the National Institute of Public Health, Cuernavaca, Mexico; 4 Department of Emergency Medicine, Johns Hopkins School of Medicine, Baltimore, MD, USA; 5 The author was affiliated with the Department of Emergency Medicine, Aga Khan University, Karachi, Pakistan at the time when study was conducted

**Keywords:** poisoning, emergency department, Pakistan, injury

## Abstract

**Background:**

Acute poisoning is one of the most common reasons for emergency department visits around the world. In Pakistan, the epidemiological data on poisoning is limited due to an under developed poison information surveillance system. We aim to describe the characteristics associated with intentional and unintentional poisoning in Pakistan presenting to emergency departments.

**Methods:**

The data was extracted from the Pakistan National Emergency Department Surveillance (Pak-NEDS) which was an active surveillance conducted between November 2010 and March 2011. All patients, regardless of age, who presented with poisoning to any of Pakistan's seven major tertiary care centers' emergency departments, were included. Information about patient demographics, type of poisoning agent, reason for poisoning and outcomes were collected using a standard questionnaire.

**Results:**

Acute poisoning contributed to 1.2% (n = 233) of patients with intentional and unintentional injuries presenting to EDs of participating centers. Of these, 68% were male, 54% were aged 19 to 44 and 19% were children and adolescents (<18 years). Types of poisoning included chemical/gas (43.8%), drug/medicine (27%), alcohol (16.7%) and food/plant (6%). In half of all patients the poisoning was intentional. A total of 11.6% of the patients were admitted and 6.6% died.

**Conclusion:**

Poisoning causes more morbidity and mortality in young adults in Pakistan compared to other age groups, half of which is intentional. Improving mental health, regulatory control for hazardous chemicals and better access to care through poison information centers and emergency departments will potentially help control the problem.

## Background

Acute poisoning and chemical exposure is a growing problem around the world. This can be attributed in large part due to an increasingly rapid rate of industrialization and a simultaneous increase in the number and types of chemicals available [1]. According to the Chemical Abstracts Service (CAS) Registry, more than 83 million chemical substances are currently available and approximately 4000 new chemicals are introduced in the world every day [2,3]. The abundance of such chemicals has important implications for health across the globe.

The recently published Global Burden of Disease Study (GBD) showed that worldwide, unintentional poisoning was responsible for an estimated 180,000 deaths in 2010. This translates into a mortality rate of 2.6 per 100,000 inhabitants, making poisoning a top 50 cause of death. However, compared to 1990 figures, it appears that unintentional poisoning has decreased significantly; an 11% reduction in total deaths and a 34% decrease in the mortality rate [4]. In addition, over 8.9 million disability-adjusted life years (DALYs) were lost due to poisoning in 2010, which is almost 20% less than in 1990 [5]. Despite these apparent reductions, it is important to realize that low- and middle-income countries (LMICs) account for 91% of deaths and DALYs due to unintentional poisoning [6].

Poisoning is also responsible for a significant proportion of intentional injuries, particularly those that are self-inflicted. It is estimated that 23% of self-inflicted injuries globally involve the deliberate use of pesticides [7]. However, the type of poison used for deliberate self-poisoning varies significantly by region. In LMICs, pesticides such as organophosphate, carbamate, organochlorine, paraquate and aluminium phosphide are the major poisons used, especially in rural areas, and are associated with high mortality, whereas in urban areas, medicines are more common agents and generally associated with low mortality [8]. Overall mortality due to self-poisoning in LMICs (10-20%) is much higher than in high-income countries (0.5-1%) due to toxicity of available poisoning agents and lack of emergency medical services [8].

While unintentional poisoning seems to be decreasing globally, in Pakistan this condition was the 26^th ^cause of mortality in 1990 but increased to the 22^nd ^by 2010. The GBD estimates that around 11,233 deaths occurred in 2010, which is equivalent to a mortality rate of 6.6 per 100,000 and a total of 603,885 DALYs lost [4]. The National Health Survey Pakistan 1990-1994, a nationally representative household survey, reported an incidence of 3.3 non-fatal injuries due to poisoning per 1,000 per year from which 3.4% recovered with handicap [9]. Estimates from previous reports range from 21.5% to 77.0% for non-fatal injuries; while self-inflicted poisoning ranges from 18% to 70% and homicidal poisoning from 1.8% to 5% [10,11]. Such findings highlight a large amount of uncertainty and could be due to changes in the epidemiology of poisoning, regional differences and even changes to the case definition itself. Poisons most commonly employed in Pakistan are organophosphates (found in insecticides) followed by tranquilizers and narcotics [10-14]. Among medical drugs, benzodiazepines accounted for 60% of the cases but are also featured in almost all cases of multiple drug overdoses [14].

The objective of this study was to provide evidence on the characteristics associated with intentional and unintentional poisoning in Pakistan using information from the Pakistan National Emergency Department Surveillance project (Pak-NEDS). Specifically we wanted to evaluate the frequency of poisoning, patient demographics, underlying reasons for poisoning, nature of agents, types of medical care provided, and final patient outcomes for patients presenting to tertiary care hospitals in Pakistan.

## Methods

This study uses data from a larger project conducted to determine the feasibility of establishing a Pakistan National Emergency Department Surveillance System (Pak-NEDS). This project focused on the development of a cross-sectional registry using active surveillance. Out of approximately 135 tertiary care teaching hospitals in Pakistan, seven centers with busy emergency departments were selected; Aga Khan University (AKU) and Jinnah Post-graduate Medical Center in Karachi; Benazir Bhutto Hospital in Rawalpindi; Lady Reading Hospital in Peshawar; Mayo Hospital in Lahore; Sandeman Provincial Hospital in Quetta; and Shifa International Hospital (SIH) in Islamabad. All are tertiary care urban centers; AKU and SIH are private hospitals while the rest are public hospitals. AKU was the main coordinating center for the study.

Data was collected for a period of three months in each center at different time intervals between November 2010 and March 2011. The different time intervals in these centers were mainly due to logistic arrangements and support but all centers completed the three month period. Data collectors were specifically hired and trained and worked in three shifts to provide 24/7 coverage. Data collection was conducted through patient or next of kin interviews and emergency department (ED) medical records. Data was collected on males and females of all ages who presented to participating EDs with acute poisoning. A one-page standardized data collection form was developed based on the ambulatory care survey tool of the Centers for Disease Control and Prevention, USA and previous injury surveillance work done in Pakistan [15]. The data collection form recorded patient socio-demographic characteristics such as age, gender and province. The form also recorded data on the mode of arrival (ambulance versus non ambulance), type of hospital (public versus private) intentionality of poisoning as per physician discretion, provisional diagnosis of the category of poison used, the professional level of the care provider and the type of services used (i.e. only ED, ED observation unit, hospitalization in general ward or intensive care unit), reason for discharge from the emergency department and outcome (died versus survived and admitted versus not admitted) during their stay in the ED.

Hard copies of the data collection tool were sent to the coordinating center at AKUH and entered into a database using EpiInfo version 3.3.2. Statistical analysis was done using SPSS version 19 [16,17]. Descriptive statistics (frequencies and percentages) were calculated for age, gender, province, mode of arrival, type of hospital, intent of injury, category of poison ingested, type of services used, type of care provider and reasons for discharge. Cross tabulations were done to compare poisoning outcomes (died versus survived), admission status (admitted versus not admitted) and intentionality (intentional versus unintentional poisoning) for all the above mentioned variables. Differences among categories were evaluated using the Pearson chi-square test or Fisher's exact test [18]

This study was approved by the Institutional Review Board at all participating sites. Verbal informed consent was obtained from all the study participants and no patient identifiers were collected in the study.

## Results

Poisoning represented 1.2% (n = 233) of patients with intentional and unintentional injury (n = 19102) presenting to the EDs of the participating tertiary care centers across Pakistan during the study period. Complete demographic information was available for around 92% of the subjects (Table 1). The majority (41%) of patients were between 25 - 44 years of age; teenagers and young adults (15 - 24 years) made up a third of the subjects, while males comprised 68% of all patients. This study was carried out in all four provinces of Pakistan.

**Table 1:** Characteristics and outcomes of acute poisoning patients in Pakistan from November 2010 to March 2011 in seven major tertiary care Emergency Departments of Pakistan

Variables	n	%
**Age (n = 211)**		
< 5 years	4	1.9
5-14 years	21	10.0
15-24 years	58	27.5
25-44 years	87	41.2
45-64 years	34	16.1
65+ years	7	3.3

**Gender (n = 217)**		
Male	148	68
Female	69	31.8

**Mode of arrival (n = 197)**		
Ambulance	14	7.1
Non ambulance	183	92.8

**Type of hospital (n = 233)**		
Public hospital	223	95.7
Private hospital	10	4.3

**Intent of injury (n = 135)**		
Unintentional	68	50.4
Self-poisoned	26	19.3
Poisoned by someone else	41	30.4

Types of services used and reasons for discharge* (n = 181)		
Only ED (proper discharge)	63	34.8
Ward admission	20	11.0
ICU/HDU admission	1	.6
Observation unit	19	10.5
Follow-up	58	32.0
Died	12	6.6
Others(LAMA/LWBS/Referred out)	8	4.4

Type of care provider*(n = 193)		
Paramedic	84	43.5
House officer/intern	72	37.3
PG trainee/Resident	19	9.8
Medical Officer	132	68.4
Nurse/midwife	129	66.8
Attending physician	5	2.6

**Category of Poison ingested (n = 233)**		
Drug/Medicine	63	27.0
Alcohol	39	16.7
Chemicals/Gases and Vapours	102	43.8
Food/Plants	14	6.0
Others	15	6.4

LAMA = Left against medical advice

ICU/HDU= Intensive care unit/High Dependency Unit

LWBS = Left without being seen

*(Multiple response variable)

From the 197 subjects for whom data on mode of arrival was present, 93% arrived via non-ambulance transport, and 7% were brought by an ambulance (Table 1). Patients presenting to public hospitals represented 96% of the study population. Most of the cases suffered from chemical, gas and vapor poisoning (44%), while poisoning from drug or medical drug overdose was the second-leading type (27%). Of the data available for 193 patients, only 3% were seen by an attending physician, while 79% were seen by a post-graduate trainee or medical officer (Table 1). It is possible that a patient was seen by more than one type of care provider, therefore this category is not mutually exclusive.

Information on the type of services utilized and reasons for discharge was available for 78% (181/233) of all subjects (Table 1). Most patients (67%) were safely discharged from the ED, and around half of these were asked to come for a follow-up visit. 11% were kept in an observation unit, while another 11% were admitted to the ward. One patient was admitted to the ICU, and twelve patients died as a result of poisoning; of these, two were dead on arrival.

When examining subjects by survival, 52 cases did not have information about outcome (either dead or alive). Out of 181, the case fatality rate was 6.63% overall (16.67% intentional; 1.69% unintentional; intentionality unknown in 1.61%). Most of the deaths i.e. 50% (5/10) took place among those aged 25-44 years, and the majority (90%; 9/10) were male (Table 2). Of the twelve deaths recorded, all deaths occurred in public hospitals, and most (n = 8) were due to poisoning by drug abuse or overdose of therapeutic drugs. Differences between those who died and those who survived were statistically significant only when comparing the category of poison ingested (P-Value = 0.031); those who died tended to use more drugs/medicine (66%) compared to those who survived who most commonly were exposed to chemical/gas/fumes (47%).

**Table 2:** Comparison of poisoning patient characteristics based on patient disposition from November 2010 to March 2011 in seven major tertiary care Emergency Departments of Pakistan

Variables	Survived, N = 169	Died, N = 12	p-value	Discharged from ED, N = 121	Admitted, N = 40	p-value
				
	n (%)	n (%)		n (%)	n (%)	
**Age Group**	**163**	**10**	0.09	**117**	**39**	0.048
< 5 years	3 (1.8)	0 (0)		1 (0.9)	2 (5.1)	
5-14 years	17 (10.4)	1 (10)		16 (13.7)	1 (2.6)	
15-24 years	50 (30.7)	0 (0)		37 (31.6)	10 (25.6)	
25-44 years	66 (40.5)	5 (50)		41 (35)	21 (53.8)	
45-64 years	21 (12.9)	4 (40)		18 (15.4)	3 (7.7)	
65+ years	6 (3.7)	0 (0)		4 (3.4)	2 (5.1)	

**Gender**	**168**	**10**	0.178	**121**	**40**	0.230
Male	114 (67.9)	9 (90)		85 (70.2)	24 (60)	
Female	54 (32.1)	1 (10)		36 (29.8)	16 (40)	

**Mode of arrival**	**158**	**4**	0.267	**114**	**37**	0.731
Ambulance	11 (7.0)	1 (25.0)		8 (7.0)	3 (8.1)	
Non ambulance	147 (93.0)	3 (75.0)		106 (93.0)	34 (91.9)	

**Type of hospital**	**169**	**12**	1.000	**121**	**40**	<0.001
Public hospital	159 (94.1)	12 (100)		120 (99.2)	31 (77.5)	
Private hospital	10 (5.9)	0 (0)		1 (0.8)	9 (22.5)	

**Category of poison ingested**	**169**	**12**	0.031	**121**	**40**	0.681
Drug/Medicine	39 (23.1)	8 (66.7)		28 (23.1)	8 (20)	
Alcohol	27 (16)	2 (16.7)		20 (16.5)	7 (17.5)	
Chemical/Gas/Fumes	80 (47.3)	2 (16.7)		53 (43.8)	22 (55)	
Food/Plants	10 (5.9)	0 (0)		9 (7.4)	1 (2.5)	
Others	13 (7.7)	0 (0)		11 (9.1)	2 (5)	

Around 54% of those admitted were between the ages of 25 and 44, with a male to female ratio of 3:2 (Table 2). Of patients admitted, Almost 92% arrived by non-ambulance transport, and most admissions were in public hospitals. Around half of the hospital admissions were due to poisoning by chemicals, gas or fumes.

The intent of injury was recorded for only 58% of the cases, with the percentages of intentional versus unintentional poisoning approximately equal (49.6% versus 50.4%). Surprisingly, a large proportion (30% of the total for which intent is reported) were listed as poisoned by others, while 19% of the cases were self-inflicted (Table 3). For those aged 5 to 14 years, 67% (n =10) were unintentional poisoning, and 33% (n = 5) were intentional. More than three-fourths of all intentional poisoning were males. Public hospitals received all cases of intentional poisoning while non medicinal chemicals, gases and vapours were the preferred form of intentionally-used poison. Out of a total of 12 deaths, ten were poisoned by self-harm or assault, while only one was unintentional (intent was missing for one subject).

**Table 3:** Comparison of patient characteristics based on intent of poisoning from November 2010 to March 2011 in seven major tertiary care Emergency Departments of Pakistan (n = 233)

Variables	Intentional N = 67		Unintentional N = 68 n (%)	Intent not reported N = 98 n (%)	Chi Square p-value
				
	Self-inflicted N = 26 n (%)	Assault N = 41 n (%)			
**Age Group (n = 211)**					0.254
< 5 years	0 (0.0)	1 (2.5)	1 (1.6)	2 (2.4)	
5-14 years	2 (8.0)	3 (7.5)	10 (15.9)	6 (7.2)	
15-24 years	4 (16.0)	11 (27.5)	19 (30.2)	24 (28.9)	
25 - 44 years	13 (52.0)	18 (45.0)	27 (42.9)	29 (34.9)	
45 - 64 years	5 (20.0)	6 (15.0)	3 (4.8)	20 (24.1)	
65+ years	1 (4.0)	1 (2.5)	3 (4.8)	2 (2.4)	

**Gender (n = 217)**					0.13
Male	20 (80)	32 (78)	45 (67.2)	51 (60.7)	
Female	5 (20)	9 (22)	22 (32.8)	33 (39.3)	

**Mode of arrival (n = 197)**					<0.001
Ambulance	2 (11.8)	9 (22.5)	1 (1.6)	2 (2.6)	
Non ambulance	15 (88.2)	31 (77.5)	62 (98.4)	75 (97.4)	
					

**Time from event to ED presentation (Median) (n = 143)**	50 minutes (n = 12)	40 minutes (n = 31)	47 minutes (n = 51)	420 minutes (n = 49)	-

**Type of hospital**					0.047
Public hospital	26 (100)	41 (100)	61 (89.7)	95 (96.9)	
Private hospital	0 (0.0)	0 (0.0)	7 (10.3)	3 (3.1)	

**Category of poison ingested**					0.010
Drug/Medicine	10 (38.5)	8 (19.5)	20 (29.4)	25 (25.5)	
Alcohol	10 (38.5)	8 (19.5)	9 (13.2)	12 (12.2)	
Chemical/Gases and Vapours	3 (11.5)	17 (41.5)	30 (44.1)	52 (53.1)	
Food/Plants	0 (0.0)	4 (9.8)	6 (8.8)	4 (4.1)	
Others	3 (11.5)	4 (9.8)	3 (4.4)	5 (5.1)	

**Outcome (n = 181)**					<0.001
Died	8 (38.1)	2 (5.1)	1 (1.7)	1 (1.6)	
Alive	13 (61.9)	37 (94.9)	58 (98.3)	61 (98.4)	

Type of care provider* (n = 193)					
Paramedic	4 (22.2)	10 (26.3)	41 (60.3)	29 (42)	<0.01
House officer/intern	13 (72.2)	29 (76.3)	9 (13.2)	21 (30.4)	<0.001
PG trainee/Resident	0 (0.0)	6 (15.8)	9 (13.2)	4 (5.8)	0.132
Medical Officer	10 (55.6)	20 (52.6)	57 (83.3)	45 (65.2)	0.004
Nurse/midwife	15 (83.3)	33 (86.8)	37 (54.4)	44 (63.8)	0.002

*multiple response variable

There were statistical differences on the mode of arrival: a large proportion of unintentional poisoning cases arrived via non-ambulance transport (98%), while the majority of self-inflicted and assault cases arrived by ambulance (12 and 23% respectively, P-Value<0.001). The preferred poison of those self-inflicting poisoning was both drug/medicines and alcohol (39% each); while 42% of assaults and 44% of unintentional poisoning cases used chemicals/gases and vapours. There were also statistical differences in the type of care provider depending upon intentionality of poisoning. A greater proportion of unintentional cases received attention by paramedics and medical officers, while more intentional cases received care provided by house officer/intern and nurse/midwife (Table 3). We also found statistically significant differences in terms of mortality. Intentional poisonings had a higher mortality rate (16.6%) while unintentional poisonings had a mortality rate of 1.6%

## Discussion

This is the first study to document the frequency and type of poisoning presenting to multiple EDs across Pakistan. Findings highlight the fact that acute poisoning contributes to morbidity and mortality in Pakistan and specifically to a higher proportion of teenagers and young adults compared to other age groups. Under the assumption of no temporal variability in cases, the findings from this study suggest that the estimated number of poisoning cases presenting to EDs could be 932 cases/year or 2.5 cases per day in these centers. However, this is likely a gross underestimation of the country's incidence of poisonings, since only seven centers were included. At the time of this study there were 75 medical and dental council accredited institutes and 135 associated teaching hospitals in Pakistan [19] and one of the tertiary care centers in Punjab that had an affiliated poison control center did not participate in this study. Additionally, intentional poisonings are a medicolegal cases, and socially unacceptable in Pakistan. As such, individuals tend to avoid presenting to a hospital for fear of reporting and the social stigma [20].

It is important to note that most poisoning cases occurred among those aged 25 to 44 years, who are also considered the most economically active and productive members of society. Similarly, most deaths also occurred in this age-group. In contrast, according to 2010 Global Burden of Disease estimates from Pakistan, the mortality goes up with age and is highest in the elderly group [21].

This study also highlights that mortality is high and main reason is prescription drugs. The case fatality rate of 6.6% found in this study falls on the higher side of the 2.5 to 11% case fatality rates reported by other hospitals in Pakistan [10-14,22-24]. These findings also fall on the higher side of case fatality rates seen in neighboring countries like Sri Lanka (3.2%) and India (11%) [25,26]. However, this is a stark contrast with 2012 case fatality rate of 0.05% seen in the United States for exposure-related poisoning deaths [27]. Although pesticides are generally known to be more lethal and common as a poisoning agent than medical drugs [28], we could not find this as a cause of higher mortality in our study.

The fact that poisoning by chemical, gas or vapours was found to be the most common poisoning in this study might be due to various factors. Poor regulatory control on hazardous chemicals in Pakistan and lack of awareness and improper labeling of products could be underlying factors [29]. In a 2012 press release from the one of Pakistan's poison control centers, chemical poisoning was found in around 71% of all patients brought to the National Poison Control Center between 2007 and 2010 [13].

We also found that ambulance as a mode of transport was used in only 7% of total cases and in 25% of those who died. Although we could not find a statistically significant difference in terms of outcome, this could have important implications. Poisoning requires a decontamination procedure to be done as early as possible, such as gastric lavage or, charcoal decontamination. These procedures are generally more effective if done within one hour [30]. However, access to care and pre-hospital care is one of the problems faced by our country, thus the window period of one hour is lost by the time patient presents to the hospital. Ambulance services are not only limited but also have limited provision for pre-hospital care [31]. In a press release, experts in emergency medicine stressed the need for improved pre-hospital emergency medical services [32].

This study has highlighted the fact that most deaths occurred as a result of poisoning by drug abuse and overdose of therapeutic drugs, which is also a prominent cause of poisoning in high income countries [28]. However, pesticides have been previously reported to take more lives. For example, from 1999 to 2002, data from Pakistan's National Poison Control Center found that 41% cases were due to organophosphate poisoning, with a case fatality of 5% [33]. This is in contrast to findings from this study which suggest that other factors could account for such variation, including different levels of access to tertiary centers, the possibility of death before reaching a hospital and being seen at another type of medical facility.

Findings from this study have important implications for policy and the development of appropriate poisoning interventions. For example, the burden of poisoning among relatively young people combined with the large number of intentional poisonings suggests that more research is warranted as to why this is occurring. Both self-inflicted poisonings and ones that are a form of interpersonal assault, indicate that interventions focused on mental health may well be warranted in Pakistan. Additionally, this showed that most cases were managed by medical officers and interns, who generally have limited training and knowledge in medical toxicology. Clinical toxicology is a subject which is not taught in medical school or during any residency training other than emergency medicine programs or toxicology fellowships [34]. Emergency medicine is a new specialty in Pakistan with limited training centers and clinical toxicology is still an unrecognized specialty in Pakistan. This highlights the need to staff emergency departments with well-trained emergency physicians. Poison control centers with telephonic advice services should be readily available to handle poisoning cases with confidence [29]. In addition, ED counseling for attempted suicide might be promoted in these hospitals as a preventive intervention.

This study has some limitations. In most cases, data was missing for time from event to ED presentation. Such information is an important factor in timely treatment and survival and would allow for better insight into observed subject outcomes. This study was also unable to collect data on availability and use of decontamination procedures like gastric lavage, charcoal and antidotes. More than 90% of cases came from public hospitals, making comparisons between public and private hospitals with regards to treatment and outcome impossible. Additionally, data on the type of poisoning agent was collected in an aggregated way and information on specific types of poisoning could not be further analyzed. All the sites were urban tertiary care centers, which may lead to selection bias and gives little insight into the burden of poisoning in rural areas. We could not calculate true population burden of poisoning as only 7 out of 135 teaching hospitals were included for a limited period of time. This led to small sample sizes and contributed to low numbers in many cells in cross tabulation. Finally, we could not assess seasonal variation in poisoning incidents due to the limited duration of the study and logistical support available. Further studies are needed to explore temporal variability in terms of intentional and unintentional poisoning.

## Conclusion

Intentional and unintentional poisoning contributes to morbidity and mortality in Pakistan especially among young adults. Non-medicinal chemicals are the leading cause of poisoning presenting to tertiary EDs. Some of the strategies to control this problem in the future involve improved regulatory controls for hazardous chemical availability and access, improving information systems such as the establishment of a telephone advice service through poison control centers and more training in emergency medicine and clinical toxicology.

## Competing interests

The authors declare that they have no competing interests.

## Authors' contributions

NUK and NS developed the initial draft. NUK was involved in data analysis. AF was involved in data management and statistical analysis. RPN, URK, NN, JAR and AAH critically reviewed the draft. The final draft was approved by all authors
